# Zinc-Finger Nuclease Knockout of Dual-Specificity Protein Phosphatase-5 Enhances the Myogenic Response and Autoregulation of Cerebral Blood Flow in FHH.1^BN^ Rats

**DOI:** 10.1371/journal.pone.0112878

**Published:** 2014-11-14

**Authors:** Fan Fan, Aron M. Geurts, Mallikarjuna R. Pabbidi, Stanley V. Smith, David R. Harder, Howard Jacob, Richard J. Roman

**Affiliations:** 1 Department of Pharmacology and Toxicology, University of Mississippi Medical Center, Jackson, Mississippi, United States of America; 2 Human and Molecular Genetics Center, Medical College of Wisconsin, Milwaukee, Wisconsin, United States of America; 3 Department of Physiology and Cardiovascular Research Center, Medical College of Wisconsin, Milwaukee, Wisconsin, United States of America; University Medical Center Utrecht, Netherlands

## Abstract

We recently reported that the myogenic responses of the renal afferent arteriole (Af-Art) and middle cerebral artery (MCA) and autoregulation of renal and cerebral blood flow (RBF and CBF) were impaired in Fawn Hooded hypertensive (FHH) rats and were restored in a FHH.1^BN^ congenic strain in which a small segment of chromosome 1 from the Brown Norway (BN) containing 15 genes including dual-specificity protein phosphatase-5 (Dusp5) were transferred into the FHH genetic background. We identified 4 single nucleotide polymorphisms in the Dusp5 gene in FHH as compared with BN rats, two of which altered CpG sites and another that caused a G155R mutation. To determine whether Dusp5 contributes to the impaired myogenic response in FHH rats, we created a Dusp5 knockout (KO) rat in the FHH.1^BN^ genetic background using a zinc-finger nuclease that introduced an 11 bp frame-shift deletion and a premature stop codon at AA121. The expression of Dusp5 was decreased and the levels of its substrates, phosphorylated ERK1/2 (p-ERK1/2), were enhanced in the KO rats. The diameter of the MCA decreased to a greater extent in Dusp5 KO rats than in FHH.1^BN^ and FHH rats when the perfusion pressure was increased from 40 to 140 mmHg. CBF increased markedly in FHH rats when MAP was increased from 100 to 160 mmHg, and CBF was better autoregulated in the Dusp5 KO and FHH.1^BN^ rats. The expression of Dusp5 was higher at the mRNA level but not at the protein level and the levels of p-ERK1/2 and p-PKC were lower in cerebral microvessels and brain tissue isolated from FHH than in FHH.1^BN^ rats. These results indicate that Dusp5 modulates myogenic reactivity in the cerebral circulation and support the view that a mutation in Dusp5 may enhance Dusp5 activity and contribute to the impaired myogenic response in FHH rats.

## Introduction

The myogenic response is an intrinsic property of vascular smooth muscle cells (VSMC) that initiates contraction of arterioles in response to elevations in transmural pressure [1], [2] and contributes to autoregulation of renal and cerebral blood flow (RBF, CBF). [3]–[6] We recently reported that the myogenic responses of the renal afferent arteriole (Af-Art) and middle cerebral artery (MCA) and autoregulation of RBF and CBF were impaired in Fawn Hooded hypertensive (FHH) rats and were restored in a FHH.1^BN^ congenic strain in which a small segment of chromosome 1 from the Brown Norway (BN) containing 15 genes, including dual-specificity protein phosphatase-5 (Dusp5) were transferred into FHH genetic background. [7]–[9] However, the genes that contribute to the impaired myogenic response and the mechanisms involved remain to be determined.

Dusp5 is a serine-threonine phosphatase that inactivates MAPK activity[10]–[14] by dephosphorylating ERK1/2 MAP kinases [15] which modulate the activities of the large conductance Ca^2+^-activated K^+^ channel (BK) and transient receptor potential (TRP) channels. Both of these channels influence vascular reactivity and the myogenic response. [1], [16]–[19] In the present study, we found that there were 17 SNPs in the Dusp5 gene in FHH relative to BN rats. One SNP was in the 5′-UTR and three were in the coding region. Of these, two altered potential CpG methylation sites and one introduced a G155R mutation. To determine whether Dusp5 regulates vascular tone and reactivity and if the sequence variants in this gene contribute to the impaired myogenic response in FHH rats, we created and characterized a Dusp5 Zinc-finger nuclease (ZFN) knockout (KO) rat in the FHH.1^BN^ genetic background since transfer of this region of chromosome 1 containing the Dusp5 gene was shown to restore the myogenic response in cerebral arteries. We first compared the myogenic response of the MCA and autoregulation of CBF in Dusp5 KO, FHH.1^BN^ and FHH rats. We then compared the expression of Dusp5 in multiple tissues isolated from Dusp5 ZFN KO, FHH.1^BN^ and FHH rats. We also investigated whether there are differences in the expression of p-ERK1/2 in cerebral microvessels isolated from these strains as they are the primary substrates normally dephosphorylated and inactivated by Dusp5 [15], [20].

## Materials and Methods

### General

Experiments were performed on 33 FHH, 68 FHH.1^BN^ and 92 Dusp5 KO male rats bred in our in house colonies and 16 age-matched Sprague-Dawley (SD) male rats purchased from Charles River Laboratories (Wilmington, MA). The animal care facility at the University of Mississippi Medical Center is approved by the American Association for the Accreditation of Laboratory Animal Care. The rats had free access to food and water throughout the study and all protocols received prior approval by the Institutional Animal Care and Use Committees (IACUC) of the University of Mississippi Medical Center.

### Identification and confirmation of SNPs in Dusp5 in FHH versus FHH.1^BN^ rats

We first performed an *in silico* analysis of the sequence of the Dusp5 gene in FHH versus BN rats, which is publically available from the Rat Genome database (RGD, http://rgd.mcw.edu/rgdweb/report/gene/main.html?id=620854). To confirm that the SNPs identified in the database are present in our FHH and FHH.1^BN^ colonies at both the DNA and mRNA levels, we isolated genomic DNA from tail biopsies using PureLink Genomic DNA Kits (Life Technologies, Grand Island, NY) and RNA from cerebral arteries using TRIzol solution (Life Technologies, Grand Island, NY) and sequenced across the regions of interest. RNA (1 µg) was reverse transcribed using an iScript cDNA Synthesis Kit (Bio-Rad) to produce cDNA. The regions of interest were amplified in a 25 µl PCR reaction containing 25 ng of genomic DNA or 4 ng of cDNA, 25 ng of each primer, 20 mM Tris-HCl buffer (pH 8.4), 50 mM KCl, 1.5 mM MgCl_2_, 200 µM of each dNTP and 0.5 U Taq DNA polymerase (QIAGEN) using several primer pairs to cover the full length sequence. For amplification of exon 1, the following forward and reverse primers were used: 5′- AGCTTTCCGGGGCAGCGAGTG-3′ and 5′-TCAGGATACTGTGAGTAGAAG-3′. Exons 2, 3 and a portion of exon 4 that is in the codon region were amplified using the following forward and reverse primers: 5′-CGTGCTGGACCAGGGCAGCCG-3′ and 5′-GACAGAGAGAGGTCTTCAGTATTG -3'. Intronic primers were used to amplify the regions of interest from genomic DNA. Amplification of exon 1 or 2 required an additional 5 µl of Q solution since these regions were GC-rich. The PCR products were purified using a PureLink PCR Purification Kit (Life Technologies, Grand Island, NY) and then ligated into a pCR4-TOPO TA vector (Life Technologies, Grand Island, NY) by incubating at room temperature for 20 min. One Shot MAX Efficiency DH5α-T1^R^ Competent cells (Life Technologies, Grand Island, NY) were transformed using the ligated vectors according to manufacturer's instructions. The colonies were incubated at 37°C overnight in LB media with 100 µg/ml of Ampicillin. The plasmids were extracted using a QIAprep Spin Miniprep Kit (QIAGEN, Valencia, CA) and sequenced using M13 primers. The data were analyzed using ABI software (Applied Biosystems, Grand Island, NY) and compared to the BN reference sequence available on the NCBI GeneBank and RGD databases.

### Comparison of the expressions of Dusp5, p-PKC and p-ERK1/2 in FHH and FHH.1^BN^ rats

#### RT-qPCR

RNA was isolated from microdissected cerebral arteries in FHH and FHH.1^BN^ rats that was reverse transcribed as described above. Fast SYBR Green Real-Time PCR Master Mixes (Life Technologies, Grand Island, NY) which contain a blend of dTTP/dUTP that is compatible with Uracil N-Glycosylase (UNG) to eliminate DNA contamination from PCR products synthesized in the presence of dUTP were mixed with 4 ng of cDNA and 25 ng of forward (5′-CTT AAA GGT GGG TAC GAG ACC TTC TAC -3′) and reverse (5′-GAG AAT GGG CTT TCC GCA CTG -3') primers and amplified using a real-time PCR system (Mx3000P, Stratagene, La Jolla, CA). The data were analyzed with Mxpro qPCR software (Stratagene, La Jolla, CA) using the 2^-ΔΔCT^ Method [21]. The PCR products were also separated on a 1% agarose gel using a Tris-borate-EDTA (TBE) buffer visualized the intensity of the bands with 100 mg/ml of ethidium bromide (Sigma, St. Louis, MO) and analyzed using ChemiDoc MP Imaging System (Bio-Rad, Hercules, CA).

#### Western Blot

Cerebral microvessels were isolated using the Evans blue sieving procedure as previously described [22], [23] . Cerebral microvessels and brain tissue obtained from FHH and FHH.1^BN^ rats were homogenized in ice-cold RIPA buffer (R0278, Sigma-Aldrich, St. Louis, MO) in the presence of protease and phosphatase inhibitors (Cat# 88663, Thermo Scientific, Pittsburgh, PA) and the proteasome inhibitor MG 132 (Sigma-Aldrich, St. Louis, MO) [12] using a ground glass homogenizer followed by a FastPrep-24 homogenizer (MP Biomedicals, Santa Ana, CA). The homogenate was centrifuged at 1,000 g for 10 minutes at 4°C. Aliquots of supernatant protein (40 µg for cerebral microvessels and 100 µg for brain tissues) were separated on a 10% SDS-PAGE gel, transferred to nitrocellulose membranes and probed with a pan p-PKC antibody (Cat 9371, Cell signaling, Danvers, MA) at 1∶1,000 dilution. Antibodies that against p-ERK1/2 and total ERK1/2 (Cat# 4377 and Cat# 4695, Santa Cruz, Santa Cruz, CA) were used at a 1∶1,000 dilution followed by a 1∶4.000 dilution of a horseradish peroxidase (HRP)-coupled anti-rabbit secondary antibody. The membranes were then stripped and re-probed with a 1: 8,000 dilution of an anti-beta Actin antibody (ab6276, Abcam, Cambridge, MA) followed by a 1: 20,000 dilution of anti-mouse HRP-coupled secondary antibody as a loading control.

### Generation of the Dusp5 ZFN KO rats in the FHH.1^BN^ genetic background

ZFNs targeting the following sequence CAGGGCAGCCGCCACtggcaGAAGCTGCGGGAGGA in exon 1 of the rat Dusp5 gene (NM_133578) were obtained from Sigma-Aldrich (St. Louis, MO) and were used to generate a Dusp5 KO rat in the FHH.1^BN^ genetic background as previously described [24]–[26]. The ZFN mRNA was injected into the pronucleus [25], [27] of fertilized FHH.1^BN^ embryos and transferred to the oviduct of pseudopregnant females to generate Dusp5 ZFN KO founders. Tail biopsies were obtained and digested with 0.2 mg/ml proteinase K in a direct PCR lysis reagent (102-T, Viagen Biotech) at 85°C with rotation for 45 minutes. Founders were identified using the CEL-1 assay [28] and the mutations were confirmed by Sanger DNA sequencing [29]. Positive founders were backcrossed to parental strain to generate heterozygous F1 rats and the siblings were intercrossed to produce homozygous animals. Thereafter, the rats were genotyped using the following primers: Dusp5-F: 5′-GCT GCA GGA GGG CGG CGG CG -3′, Dusp5 R: 5′-CTT TAA GGA AGT AGA CCC G -3′. These primers amplified a 155 bp band for the wild type allele and a 144 bp band for the knockout allele.

### Characterization of the Dusp5 ZFN KO rats

#### Western Blot

Cerebral microvessels, liver, brain and spleen tissues were isolated from Dusp5 KO and FHH.1^BN^ rats as described above. White blood cells were also harvested using Ficoll-Paque Premium 1.084 (GE Healthcare) according to the manufacturer's protocol. A 100 µg aliquot of protein isolated from brain, liver, spleen and white blood cells (WBCs) and a 40 µg aliquot of protein isolated from cerebral microvessels was separated on a 10% SDS-PAGE gel, transferred to nitrocellulose membranes which were probed with antibodies to Dusp5 (H00001847-M04, Abnova, Taiwan; 1∶1,000) targeting AA286-384 in the C-terminus of the Dusp5 protein and antibodies raised against p-ERK1/2, total ERK1/2 and beta-Actin as described above.

#### Myogenic response on MCA

MCAs were microdissected from the brains of 9–12 week old Dusp5 KO, FHH.1^BN^, FHH and SD rats and mounted on glass micropipettes in a myograph. The bath solution was equilibrated with O_2_ (95%) and CO_2_ (5%) to provide adequate oxygenation and to maintain at pH 7.4. The diameters of the vessels were measured using a videomicrometer (VIA-100, Boeckeler Instruments) at intraluminal pressures ranging from 40 to 140 mmHg in steps of 20 mmHg as previously described. [8], [9], [30]

#### Autoregulation of CBF

CBF autoregulation were determined on 9–12 week old male Dusp5 KO, FHH.1^BN^, FHH and SD rats. The rats were anesthetized with ketamine (30 mg/kg, *i.m.*) and Inactin (50 mg/kg, *i.p.*) and were mechanically ventilated throughout experiment to maintain pO_2_ and pCO_2_ at 100 and 35 Torr, respectively. Body temperature was maintained at 37°C during experiment. Catheters (PE-50) were placed in the femoral artery and vein and the rats received an intravenous infusion of 0.9% NaCl solution at a rate of 100 µl/min to replace surgical fluid losses. The scalp was exposed and the cranial bone was thinned 3 mm lateral and 6 mm posterior to the Bregma using a handheld drill until the pial vessels became visible through the thinned window. CBF was monitored bilaterally with a laser-Doppler flow meter (PF5001, Perimed Corp, Jarfalla, Sweden). After surgery and a 30-min equilibration period, mean arterial pressure (MAP) was lowered to 90–100 mmHg by increasing the depth of pentobarbital anesthesia (1–5 mg/kg, *i.v.*). Baseline regional CBF was measured, then systemic pressure was elevated in steps of 10–20 mmHg by graded *i.v*. infusion of phenylephrine (0.5–5 µg/min). [31]–[33] MAP was maintained for 3–5 min until a new steady-state level of CBF was obtained. CBF was expressed as a percentage of the baseline laser-Doppler flow signal.

### Statistics

Mean values ± SEM are presented. The significance of the differences in the expression of various proteins and mRNA in FHH, FHH.1^BN^ and Dusp5 KO rats was determined using one-way ANOVA. The significance of the differences in mean values between and within groups in the myogenic responses and autoregulation of CBF was determined using a two-way ANOVA for repeated measures and Holm-Sidak test for preplanned comparisons. A *P* value <0.05 was considered to be statistically significant.

## Results

### Sequence analysis and expression of Dusp5, p-ERK1/2 and p-PKC in FHH and FHH.1^BN^ rats

**Figure 1:**
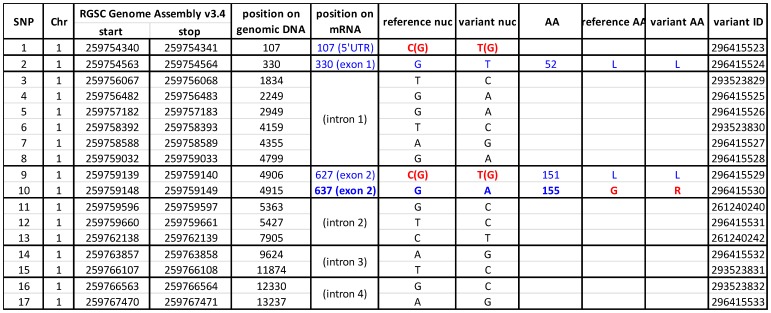
Comparison of sequence variants in the Dusp5 gene in FHH and Brown Norway (BN) rats. Analysis of the NeXT Generation sequence data available on the Rat Genome database (RGD, http://rgd.mcw.edu/rgdweb/report/gene/main.html?id=620854). These results indicate that there are 17 SNPs in the Dusp5 (NM -133578) gene in FHH/EurMcwi(variant nuc) rats as compared to Brown Norway (reference nuc) rats. Most of the SNPs are located in introns. There is a C107T SNP is located in the 5′-UTR, and three are found in the coding region including a G330T SNP in exon 1, a C627T and a G637A SNP in exon 2. The C107T SNP alters a CpG site and the C627T SNP alters one of the six 5 CpG's methylation sites previously identified in exon 2. The G637A SNP causes G155R mutation in the Dusp5 protein in FHH rats.

**Figure 2:**
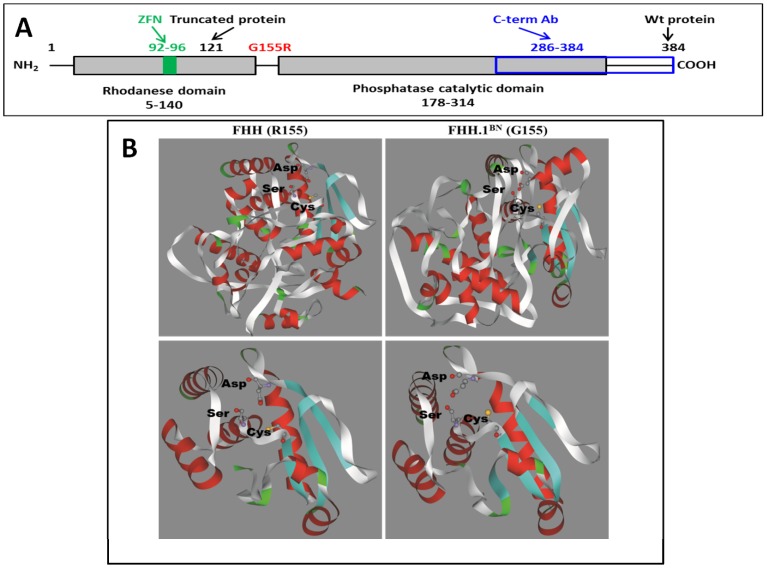
Identification of the Zn-finger target site and deletion in the Dusp5 KO strain. **Panel A** presents a schematic model of the Dusp5 protein. The Dusp5 Zinc-finger construct targets amino acids (AA) 92–96 in the N-terminal regulatory rhodanese domain (AA5-140) resulting in the introduction of a premature stop codon at AA121 that is predicted to produce a truncated protein. The Dusp5 antibody used in these studies targets AA286-384 in the C-terminal phosphatase catalytic domain (AA178-314). **Panel B** presents a comparison of I-TASSER predicted structure and the folding of the Dusp5 protein in FHH (155R) and FHH.1^BN^ (155G) rats. The upper panels show the predicted structure of the Dusp5 protein in both strains based on the complete AA sequence. The putative catalytic triad (Asp232/Ser268/Cys263) is shown in a “stick figure” form and the 3-letter AA codes are labeled in black. The rest of the protein is represented as ribbon running along the backbone. Secondary structural elements are depicted by color with helices, beta sheets and coils represented in red, cyan and white, respectively. The putative catalytic triad is magnified and shown in “stick figure” form in the lower panel and the 3-letter AA codes are labeled with in black. Only residues 174–320 of Dusp5 protein are presented in order to enhance the view of the putative catalytic triad. There are significant structural differences both in the overall folding of the Dusp5 protein that impact on the structure of the active site/catalytic triad region between the strains. This may account for the observed differences in the activity of the Dusp5 protein in FHH versus FHH.1^BN^ rats.

The results of the comparative sequence analysis are presented in **Figure 1**. We identified 17 SNPs in the Dusp5 gene in FHH versus the BN reference sequence. Most of the SNPs were in introns, however, there were four SNPs in the Dusp5 mRNA including a C107T SNP in the 5′-UTR, a G330T SNP in exon 1, a C627T and a G637A in exon 2. All of these SNPs were verified in our FHH and FHH.1^BN^ strains by sequencing cDNAs derived from mRNA extracted from the isolated cerebral vessels. The C107T SNP altered a CpG site and the C627T SNP altered one of six CpGs in exon 2 that were previously identified as methylation sites by bisulfite modification. [34]. Moreover, the G637A SNP caused a G155R mutation (**Figure 2A**) that is predicted using I-TASSER modeling package [35]–[37] may alter the folding of the protein and the active site conformation of the Dusp5 protein in FHH versus FHH.1^BN^ rats as shown in **Figure 2B** **.**

**Figure 3:**
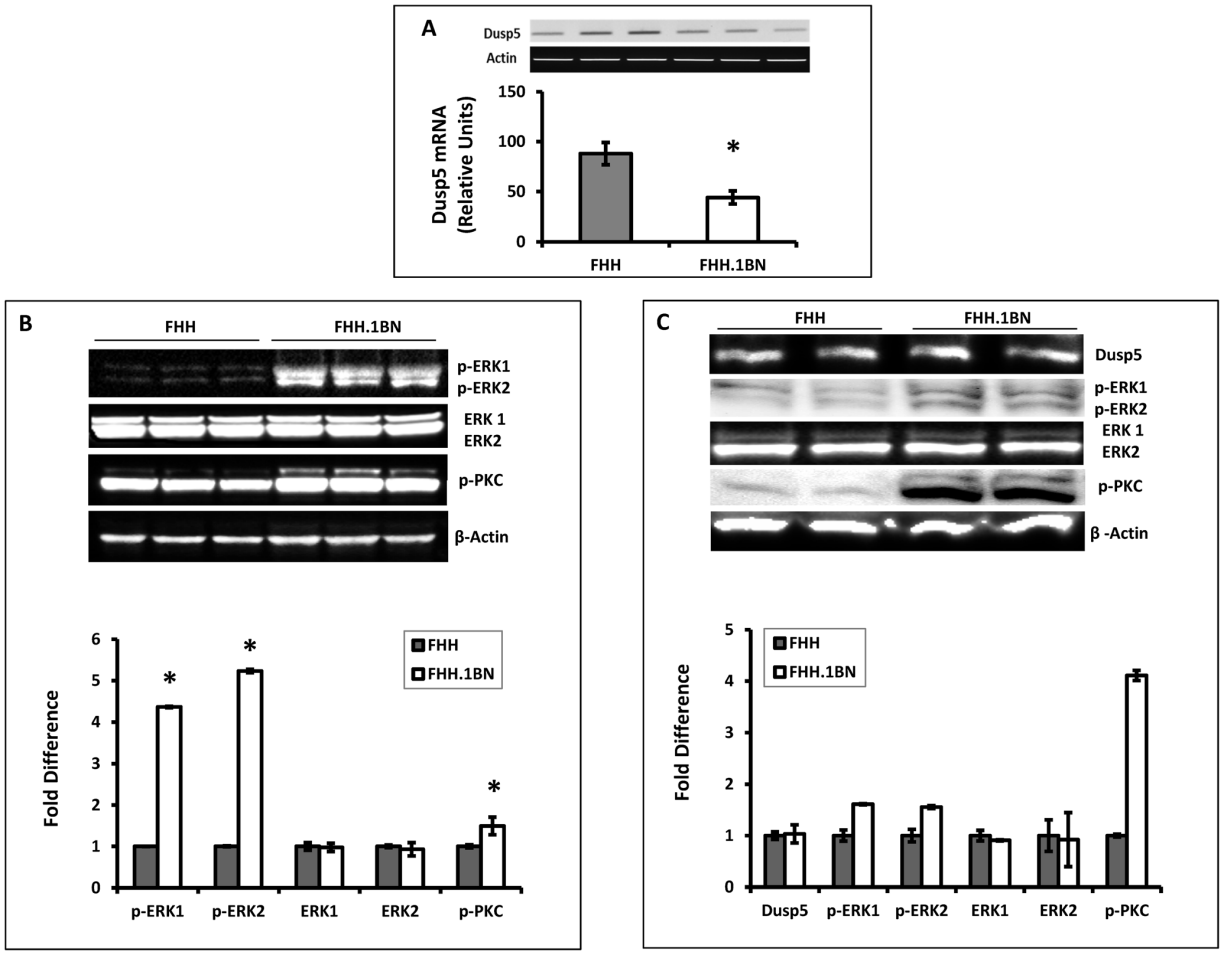
Comparison of the expression and activity of Dusp5 in FHH versus FHH.1^BN^ rats. **Panel A** presents a comparison of the expression of Dusp5 mRNA in cerebral arteries of FHH versus FHH.1^BN^ rats. The upper portion of the figure presents the representative images of gels showing the qPCR products and the bar graph below compares the expression levels. **Panel B** presents a comparison of the expression of phosphorylated-ERK1/2, total ERK1/2, phosphorylated-PKC and beta-Actin in the brain of FHH and FHH.1^BN^ rats. The upper panel presents the representative images and the lower panel presents the relative quantitation. **Panel C** presents a comparison of expression of these proteins in cerebral microvessels of FHH as compared with FHH.1^BN^ rats. All of the vessels isolated from one strain were pooled into a single sample. The upper panel presents the representative images and the lower panel presents the quantitation of the images. Mean values ± SE from 3 rats per strain are presented in Panel A and Panel B. Panel C represents the results from duplicate aliquots run from a single pooled microvessel sample isolated from 8–10 rats per strain. * indicates a significant difference from the corresponding value in FHH rats.

To determine if the C107T and C627T SNPs that altered CpG sites and the G637A SNP that caused a G155R mutation might alter the expression of Dusp5 in FHH versus FHH.1^BN^ rats, RT-qPCR and western blot experiments were performed. The results presented in **Figure 3A** indicate that the expression of Dusp5 mRNA is 2-fold higher in cerebral arteries of FHH than in the FHH.1^BN^ control rats but the expression of protein is not different between these strains (**Figure 3C**). Moreover, as presented in **Figure 3B and 3C**, the expression of p-ERK1/2, the primary substrates for dephosphorylation by Dusp5, is significantly reduced in cerebral microvessels and the brains of FHH relative to FHH.1^BN^ rats, while total ERK1/2 levels are not significantly altered. The expression of p-PKC protein is also significantly elevated in FHH.1^BN^ as compared to FHH rats.

### Generation and characterization of the Dusp5 ZFN KO rats

**Figure 4:**
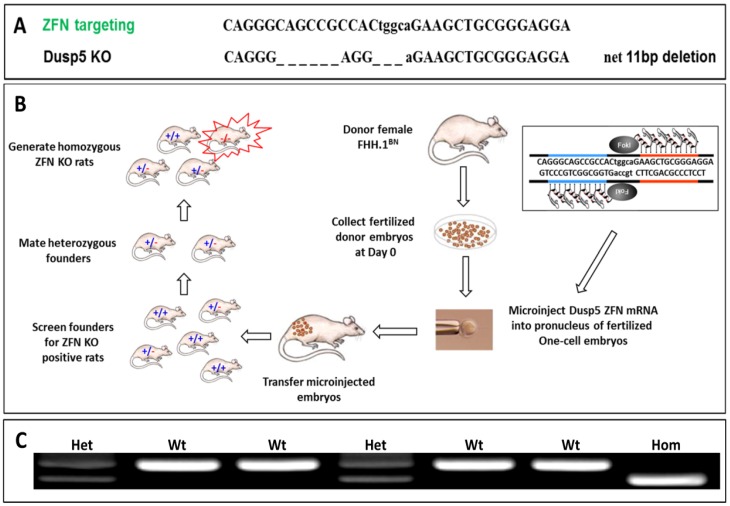
Schematic describing the generation of Dusp5 ZFN KO rats in the FHH.1^BN^ genetic background. **Panel A** presents the sequence of the Dusp5 ZFN. The Dusp5 specific ZFN introduced a 14 bp deletion and a 3 bp insertion resulting in a net 11 bp deletion between nucleotides 449–464 in the Dusp5 sequence in the KO animals that introduced a frame shift mutation. **Panel B** presents the strategy for the generation of Dusp5 ZFN KO rats in the FHH.1^BN^ genetic background. Fertilized donor embryos from female FHH.1^BN^ rats were collected and the Dusp5 ZFN mRNA was microinjected into pronuclei of the fertilized one-cell embryos. These embryos were transferred back to a foster mother. The heterozygous founders were brother-sister mated to generate the homozygous ZFN KO founders and the FHH.1^BN^ wild type control rats. **Panel C** presents an example of PCR genotyping of the region of interest in FHH.1^BN^ and Dusp5 KO rats. The rats were genotyped using the following primers: Dusp5-F: 5′-GCT GCA GGA GGG CGG CGG CG -3′, Dusp5 R: 5′-CTT TAA GGA AGT AGA CCC G-3′. These primers amplify a 155 bp band for the wild type allele and a 144 bp band for the knockout allele. Both bands are observed in heterozygous rats.

The homozygous Dusp5 knockout (KO) and wild type FHH.1^BN^ control strain were derived from an intercross of the heterozygous Dusp5 founders. Genotyping and sequencing of the Dusp5 KO strain indicated that there is a 14 bp deletion and a 3 bp insertion between nucleotides 449–464 in Dusp5 mRNA that creates a frame shift mutation which is predicted to introduce a premature stop codon at amino acid (AA) 121 (**Figure 2A** **,** **4A, 4B**). The genotypes of the animals were verified using PCR as shown in **Figure 4C**.

**Figure 5:**
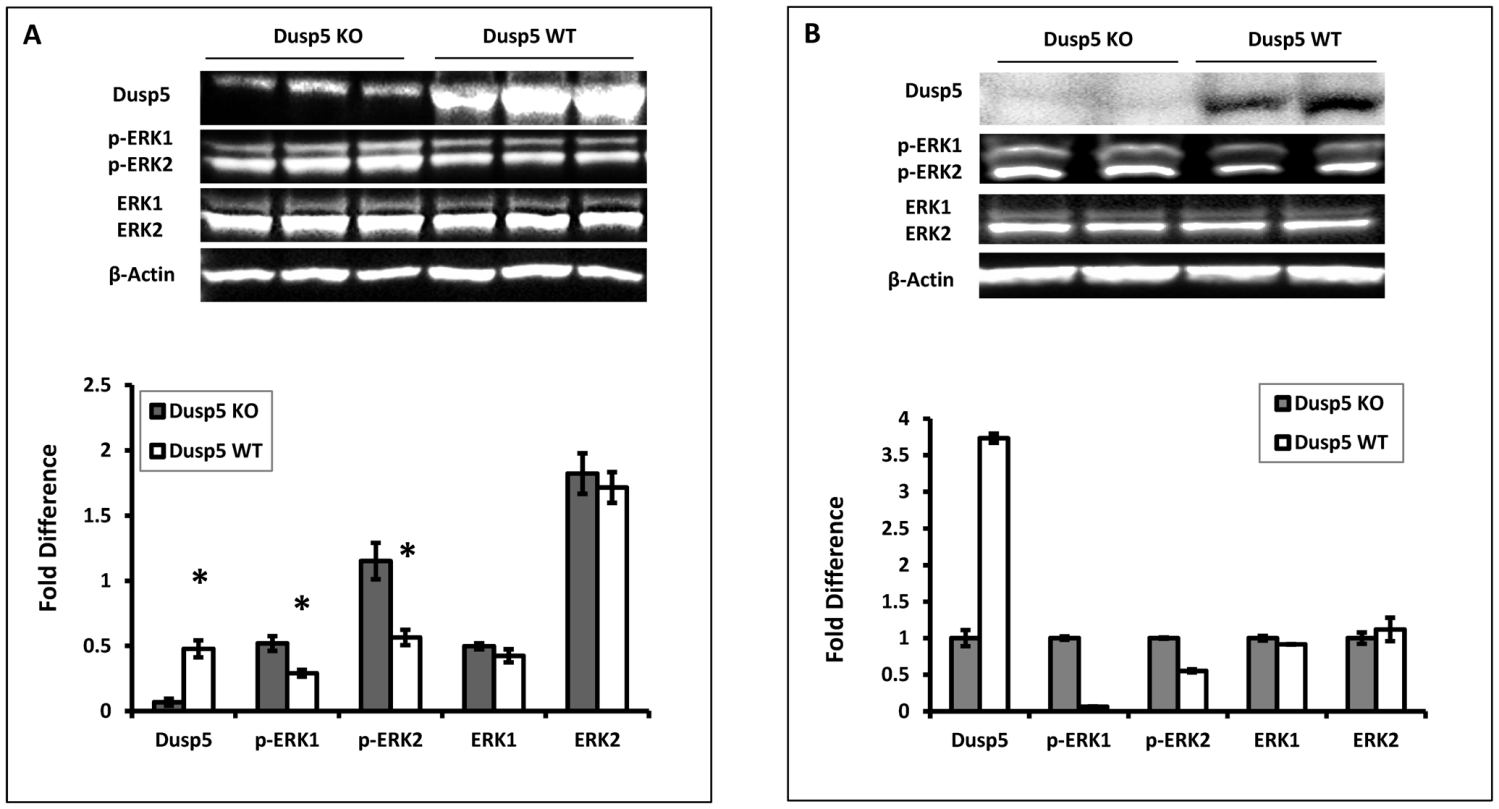
Comparison of the expression and activity of Dusp5 in WBCs isolated from Dusp5 ZFN KO versus FHH.1^BN^ rats. **Panel A**: The expression of Dusp5 protein in WBCs is nearly absent in Dusp5 KO versus FHH.1^BN^ rats. The levels of phosphorylated-ERK1/2 protein are significantly increased in Dusp5 KO compared to FHH.1^BN^ rats, but there is no change in the expression of total ERK or beta-Actin. **Panel B** presents the results of the expression of Dusp5, p-ERK1/2, total ERK1/2 and β-Actin protein from duplicate aliquots of a single pooled microvessel sample isolated from 8–10 Dusp5 KO and FHH.1^BN^ rats. The upper panel presents a representative image of the gels and the lower panel presents the quantitation of the images above. Mean values ± SE from 3 rats per strain are presented in Panel A. * indicates a significant difference from the corresponding value in Dusp5 KO rats.

Dusp5 is a ubiquitous protein that is abundantly expressed in the brain, spleen and WBCs [11], [12], [38]. The expression of Dusp5 protein in WBCs isolated from Dusp5 KO versus FHH.1^BN^ control rats was compared using an anti-Dusp5 antibody that targeted AA286-384 (**Figure 2A**) in the C-terminus of the protein to confirm that the introduction of the new stop codon produces a truncated Dusp5 protein in the KO animals. The results presented in **Figure 5** indicate that the expression of Dusp5 protein is markedly reduced in WBCs (**Figure 5A**) and cerebral microvessels (**Figure 5B**) isolated from Dusp5 KO rats relative to the FHH.1^BN^ control strain and in other tissues (brain, liver and spleen, data not shown). We also compared the expression of the primary substrates of Dusp5, p-ERK1/2, in these strains. The results presented in **Figure 5** indicate that the levels of p-ERK1/2 are enhanced, while the expression of total ERK1/2 is not significantly different in WBCs (**Figure 5A**) and cerebral microvessels (**Figure 5B**) in Dusp5 KO rats compared to FHH.1^BN^ control rats.

### Comparison of the myogenic response of the MCA of Dusp5 KO and FHH.1^BN^ rats

**Figure 6:**
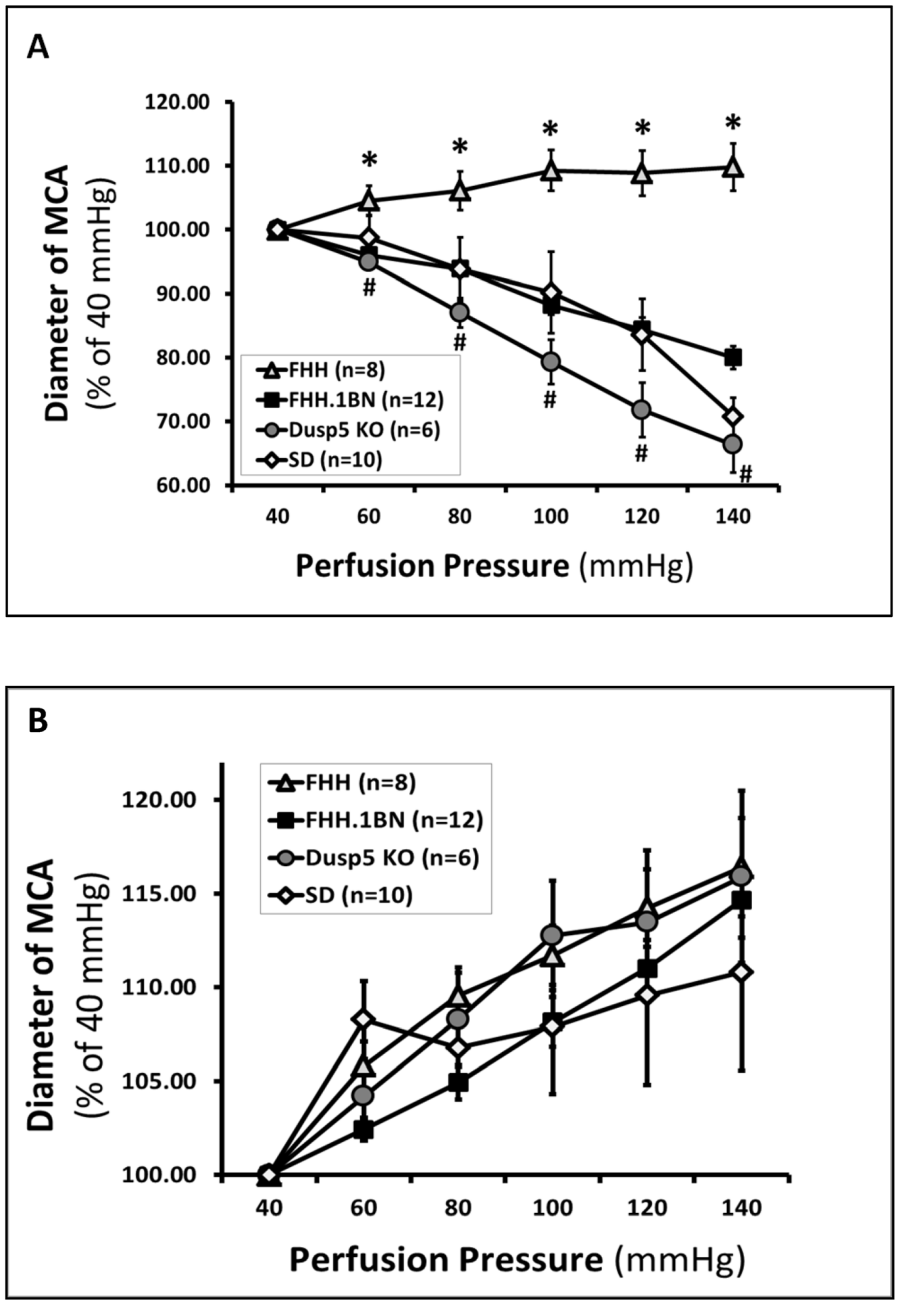
Comparison of the myogenic response in middle cerebral artery (MCA) isolated from Dusp5 ZFN KO versus FHH.1^BN^ and FHH rats. **Panel A** presents the passive pressure-diameter curves in Ca^2+^ free solution at each pressure in all strains. **Panel B**: The luminal diameter of the MCA decreased from 100 to 66±4% in Dusp5 KO rats and from 100 to 80±2% in FHH.1^BN^ rats when the perfusion pressure was increased from 40 to 140 mmHg, whereas it was dilated in FHH rats (from 100 to 110±4%). The MCA also constricted in Sprague Dawley rats that is widely used as a control strain for the myogenic response. Mean values ± SE are presented. Numbers in parentheses indicate the number of vessels studied per group. * indicates a significant difference in the corresponding value in FHH versus all the other strains. # indicates there is a significant difference between Dusp5 KO and FHH.1^BN^ rats.

The luminal diameter of the MCA in FHH.1^BN^ rats (n = 12) decreased by 20±2% when the perfusion pressure was increased from 40 to 140 mmHg. The myogenic response of MCA isolated from Dusp5 KO rats was significantly greater, and the diameter of these vessels decreased by 34±7% when the perfusion pressure was increased over the same range. In contrast, the myogenic response of the MCA of FHH rats was markedly impaired as the diameter of these vessels only increased by 10±4% when pressure was increased from 40 to 140 mmHg (**Figure 6A**). The passive diameter curves generated in Ca^2+^ free solution in all strains were not significantly different (**Figure 6B**).

### Comparison of the autoregulation of CBF of Dusp5 KO and FHH.1^BN^ rats

**Figure 7:**
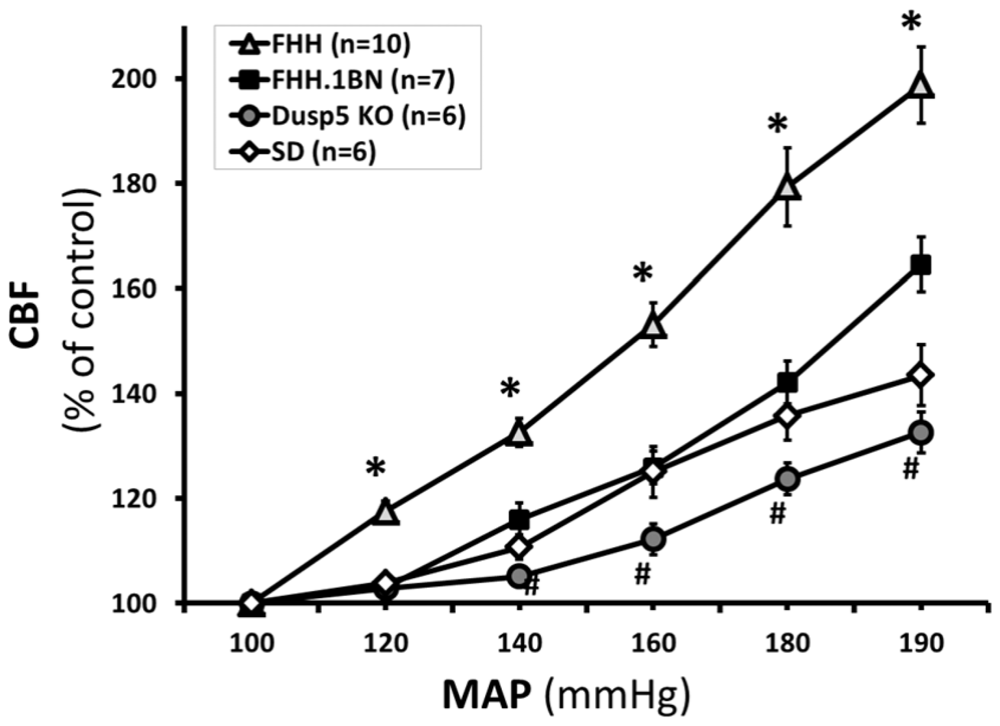
Comparison of autoregulation of CBF in Dusp5 ZFN KO versus FHH.1^BN^ and FHH rats. The relationships between cerebral blood flow and mean arterial pressures in 9–12 week old Dusp5 ZFN KO, FHH.1^BN^, FHH and Sprague Dawley rats are compared. Mean values ± SE are presented. * indicates significantly difference in the corresponding value in FHH rats versus all the other strains. # indicates a significant difference in the corresponding values in Dusp5 KO and FHH.1^BN^ rats. Numbers in parentheses indicate numbers of animal studied per strain.

Autoregulation was markedly impaired and CBF increased by 54±6% in FHH rats when MAP was increased from 100 to 160 mmHg. CBF was autoregulated to a greater extent in the FHH.1^BN^ and Dusp5 KO rats were not significant different and only increased by 26±3% and 12±3%, respectively, when MAP was increased over the same range. The range of the autoregulation of CBF since CBF rose by 33±4% when pressure was increased from 100 to 190 mmHg in Dusp5 KO rats versus an increase of 65±5% in the FHH.1^BN^ rats and 99±3% in the FHH animals (**Figure 7**).

## Discussion

We recently reported that the myogenic response of the MCA and autoregulation of CBF were markedly impaired in FHH rats and were restored in a FHH.1^BN^ congenic strain in which Chromosome 1 from the BN rats containing 15 genes was transferred into the FHH genetic background. [7]–[9] However, the gene or genes that contribute to the impairment of vascular function and the mechanisms involved still remain obscure. In the present study, we identified 17 SNPs in the Dusp5 gene in FHH versus FHH.1^BN^ rats. Most were in the intronic region, but four were in exons including a C107T SNP in the 5′-UTR, a G330T SNP in exon 1, a C627T and a G637A in exon 2. Both of the SNPs, C107T and C627T, altered CpG sites and the C637A SNP caused a G155R mutation. To determine if the altered CpG sites and/or the G155R mutation might underlie the loss of the myogenic response in FHH rats, we created Dusp5 KO rats in the FHH.1^BN^ genetic background using ZFN KO technology [25]–[27], [39]. Site specific Zn-fingers fused to *Fok I* nuclease were introduced into the pronucleus of one cell embryos to induce double-strand DNA breaks at the target site, followed by error-prone non-homologous DNA repair which resulted in a frameshift mutation and formation of a premature stop codon leading to a truncated or non-functional protein. [25]–[27], [40] The ZFNs targeted AA92-96 in the N-terminal regulatory rhodanese domain of Dusp5 protein that is the ERK1/2 binding site (**Figure 2A**). [41], [42] The Dusp5 ZFN KO strain was successfully generated and we confirmed that this strain had a net 11 bp deletion between nucleotides 449–464 in Dusp5 mRNA that introduced a frame shift mutation and a premature stop codon at AA121 (**Figure 4A** **,** **2A**). We also confirmed that the expression of Dusp5 at the protein level was nearly absent in multiple tissues including cerebral microvessels using an antibody directly targeting AA286-384 in the C-terminus that are beyond the predicted site of the newly introduced stop codon. The loss of Dusp5 protein in the KO animals was associated with an expected increase in p-ERK1/2 levels in various tissues and cerebral microvessels compared to FHH.1^BN^ wild type animals because Dusp5 is a serine-threonine phosphatase that inactivates MAPK activity [10]–[14] by specifically dephosphorylating p-ERK1/2 MAP kinases. [15], [38]

An increase in the dephosphorylation of p-ERK1/2 by Dusp5 phosphatase would be expected to modulate BK and TRP channel activities [1], [16]–[19] and downregulate PKC, Rho/ROCK[20], [43] and STAT pathways[11] which are regulated by the MAP kinase system. Activation of BK and TRP channel activities alter the myogenic response of small blood vessels by modulating calcium entry in VSMCs. [1], [19], [22] Discovered over 100 years ago by Bayliss, the myogenic response is an intrinsic property of VSMC that initiates contraction of arterioles in response to elevations in transmural pressure. [1], [2] It is impaired following cerebral vasospasm, stroke or traumatic brain injury and the autoregulatory range is shifted to higher pressures in hypertension [3], [4], [44]–[46]. Autoregulation of CBF is one of the major mechanisms to protect the brain from elevations in perfusion pressure that promote vascular leakage and swelling of the brain [9], [19], [46]_ENREF_64. The myogenic response of MCA plays a major role in autoregulation of CBF and contributes about 50% to overall compensation to elevations in perfusion pressure [47].

In the present study, we found that the myogenic response of the MCA was greater in Dusp5 KO animals than in wild type controls and FHH rats. There was no difference in the passive pressure diameter relationships measured in Ca^2+^ free solution between these strains. The increased myogenic response was associated with enhanced autoregulation of CBF in response to elevations in systemic pressure from 100–160 mmHg in the Dusp5 KO compared to FHH.1^BN^ and FHH strains. Moreover, the range of effective autoregulation of CBF was extended to higher pressures in the Dusp5 KO rats versus FHH.1^BN^ and FHH animals. Our findings are consistent with the results of a recent study by Wickramasekera, *et al* demonstrating that downregulation of the expression of Dusp5 by siRNA in cultured cerebral arteries enhanced pressure-dependent myogenic constriction [20]. Together, these findings confirm that alteration in the expression or activity of Dusp5 modulates the myogenic response of the MCA *in vitro* and autoregulation of CBF *in vivo*.

We also examined whether the sequence variants we identified in the Dusp5 gene in FHH versus FHH.1^BN^ rats might contribute to the impaired myogenic response and autoregulation of CBF in FHH rats by altering the expression of Dusp5 and its phosphatase activity. Our RT-qPCR results indicated that the expression of Dusp5 at the message level in microdissected cerebral arteries was 2-fold higher in FHH relative to FHH.1^BN^ rats, but the expression at protein level was not significantly different. This suggests that the difference in the myogenic response of MCA between FHH and FHH.1^BN^ rats is not due to changes in the expression of Dusp5 secondary to the two SNPs (C107T and C627T) in the Dusp5 gene in FHH rats that is predicted to alter CpG sites that may possibly cause DNA demethylation and alter transcriptional activity. [34] However, we found that p-ERK1/2 levels were significantly decreased in FHH compared to FHH.1^BN^ rats. Although more work will be needed to rigorously test this hypothesis, a decrease in p-ERK1/2 levels is entirely consistent with the observed reduction in the myogenic response in the MCA and autoregulation of CBF observed in FHH rats. Moreover, we also found that a G637A SNP causes a G155R mutation in Dusp5 protein in FHH rats. This G155R mutation localized between the N-terminal regulatory rhodanese domain and the C-terminal phosphatase catalytic domain (**Figure 2A**) converts a nonpolar amino acid Glycine (G) to a basic polar Arginine (R) and is predicted by the I-TASSER program [35]–[37] to affect the folding of the Dusp5 protein and the conformation of the active site. **Figure 2B** illustrates the changes in the folding based on theoretical structural models (upper panel) and the expanded view presents the confirmation of the active/catalytic site (lower panel). These differences in global folding and active site conformation in FHH compared to FHH.1^BN^ rats might lead to differences in protein stability, interactions with binding partners, catalytic efficiency or catalytic activity.

An impaired myogenic response and autoregulation of CBF has been reported in various pathological conditions in patients and experimental animals including: in subarachnoid hemorrhage (SAH), [48]–[53] ischemic stroke [3], [54]–[56] and traumatic brain injury. [57]–[60] In hypertensive patients, impaired autoregulation of CBF accelerates the development of a cognitive decline. [61] However, the mechanisms involved have been difficult to directly study due to lack of an animal model in which autoregulation of CBF is altered. The present findings indicating that the myogenic response in the cerebral arteries and autoregulation of CBF are impaired in FHH rats and restored in FHH.1^BN^ congenic strain and are enhanced in our newly generated Dusp5 ZFN KO rats now fill this knowledge gap and provide an important new model system to study the mechanisms by which genetic defects in myogenic mechanisms contribute to the development of small vessel disease and brain damage.

## Perspectives and Significance

The present study reports on the creation of a Dusp5 KO rat and provides the first *in vivo* evidence that Dusp5 plays an important role in modulating the myogenic response of cerebral arteries and autoregulation of CBF. We identified a G155R mutation that might contribute to an increase in Dusp5 phosphatase activity and reduced the phosphorylation of ERK1/2 that is consistent with the impaired myogenic response and autoregulation of CBF in FHH rats. Our newly generated Dusp5 KO rat model also provides the scientific community a new model to investigate the mechanisms of impaired myogenic response in FHH rats, and the essential role of Dusp5 in the regulation of MAP kinase activity in vascular reactivity, immune response, cell proliferation and apoptosis and cancer.
